# Predictors of Motivation to Learn Among Middle-Aged and Older Men in the United States

**DOI:** 10.1093/geroni/igaa057.1439

**Published:** 2020-12-16

**Authors:** Taka Yamashita, Roberto Millar, Shalini Sahoo, Thomas Smith, Phyllis Cummins

**Affiliations:** 1 University of Maryland, Baltimore, Maryland, United States; 2 Graduate Student, Baltimore, Maryland, United States; 3 University of Maryland, Baltimore, Elkridge, Maryland, United States; 4 Northern Illinois University, DeKalb, Illinois, United States; 5 Miami University, Oxford, Ohio, United States

## Abstract

Middle-aged and older men are less likely than women to participate in adult education and training (AET) outside of their work. AET is known to provide psychological, social and economic (e.g., job-related skills) benefits throughout the life course. Research has shown that motivation to learn (MtL) is the key to promoting AET. The objective of this study is to identify MtL predictors among middle-aged and older men in the U.S. Nationally representative data (n = 1,450) of men aged 45 years and older were obtained from the 2012/2014 Program for International Assessment of Adult Competencies (PIAAC). Structural equation models were constructed to examine how a latent MtL construct measured by four 5-point Likert-type itemsMtL might be predicted by participant characteristics. Results showed that having a postsecondary degree (vs. high school or less; b = 0.19, p < 0.05), higher literacy skills (0-500 points; b = 0.01, p < 0.05), at least one parent/guardian with a postsecondary degree (vs. those without; b = 0.08, p < 0.05) and better self-rated health (b = 0.14, p < 0.05) were associated with greater MtL. Additionally, Black (b = 0.22, p < 0.05) and Hispanic (b = 0.19, p < 0.05) men showed greater MtL than White men. Overall, socioeconomic status indicators and race/ethnicity were linked to MtL. Given the known challenges involved in middle-aged and older men’s participationin AET (e.g., low and short-term participation), enhancing MtL may have long-term implications. Theoretical explanations and possible policy implications are evaluated.

